# Investigating suicide related behaviours across sexual orientation and neighbourhood deprivation levels: A cohort study using linked health administrative data

**DOI:** 10.1371/journal.pone.0282910

**Published:** 2023-03-29

**Authors:** Karanpreet Kaur Azra, Andrew Nielsen, Chungah Kim, Gabriel John Dusing, Antony Chum

**Affiliations:** 1 Department of Applied Health Sciences, Brock University, St. Catharines, Ontario, Canada; 2 Canadian Institute for Health Information, Toronto, Ontario, Canada; 3 School of Kinesiology and Health Science, York University, Toronto, Ontario, Canada; 4 Dalla Lana School of Public Health, University of Toronto, Toronto, Ontario, Canada; Kurdistan University of Medical Sciences, ISLAMIC REPUBLIC OF IRAN

## Abstract

**Background:**

There have been no studies examining how neighbourhood deprivation modifies the effects of sexual minority status on suicide-related behaviours (SRB). Sexual minority individuals in deprived areas may face unique challenges and stressors that exacerbate their risk of SRB. This study aims to investigate the association between sexual minority status and clinical SRB, and examine whether the effect of neighbourhood deprivation differs across sexual orientation.

**Methods:**

A population-representative survey sample (169,090 respondents weighted to represent 8,778,120 individuals; overall participation rate 75%) was linked to administrative health data in Ontario, Canada to measure SRB-related events (emergency department visits, hospitalizations, and deaths) from 2007 to 2017. Neighbourhood-level deprivation was measured using the Ontario Marginalisation index measure of material deprivation at the dissemination area level. Discrete-time survival analysis models, stratified by sex, tested the effects of neighbourhood deprivation and sexual minority status, while controlling for individual-level covariates.

**Results:**

Sexual minority men had 2.79 times higher odds of SRB compared to their heterosexual counterparts (95% CI 1.66 to 4.71), while sexual minority women had 2.14 times higher odds (95% CI 1.54 to 2.98). Additionally, neighbourhood deprivation was associated with higher odds of SRB: men in the most deprived neighbourhoods (Q5) had 2.01 times higher odds (95% CI 1.38 to 2.92) of SRB compared to those in the least deprived (Q1), while women had 1.75 times higher odds (95% CI 1.28 to 2.40). No significant interactions were observed between sexual minority status and neighbourhood deprivation levels.

**Conclusion:**

In both men and women, sexual minority status and neighbourhood deprivation are independent risk factors for SRB. Despite the lack of effect modification, sexual minorities living in the most deprived neighbourhoods have the highest chances of SRB. Future investigations should evaluate interventions and policies to improve sexual minority mental health and address neighbourhood deprivation.

## Introduction

Suicide-related behaviours (SRB) refers to both fatal and non-fatal self-inflicted injuries and poisonings [1]; they are both important elements of suicidality as non-fatal SRB have been shown to be a strong predictor of future suicide [2–5]. While there is growing evidence that neighbourhood deprivation [6–9] and sexual minority status [10,11] are independent risk factors for SRB, the interaction of these factors on SRB risk is unknown. The neighbourhood opportunity structure perspective offers an explanation for how the social, cultural, psychological, and economic dimensions of deprived neighbourhoods may increase the risk of SRB; however, the risk may be further amplified for sexual minorities [12]. Understanding the interactions between sexual minority status and neighbourhood deprivation may help develop new strategies to address SRB risk among the most marginalized individuals in our society.

**Fig 1:**
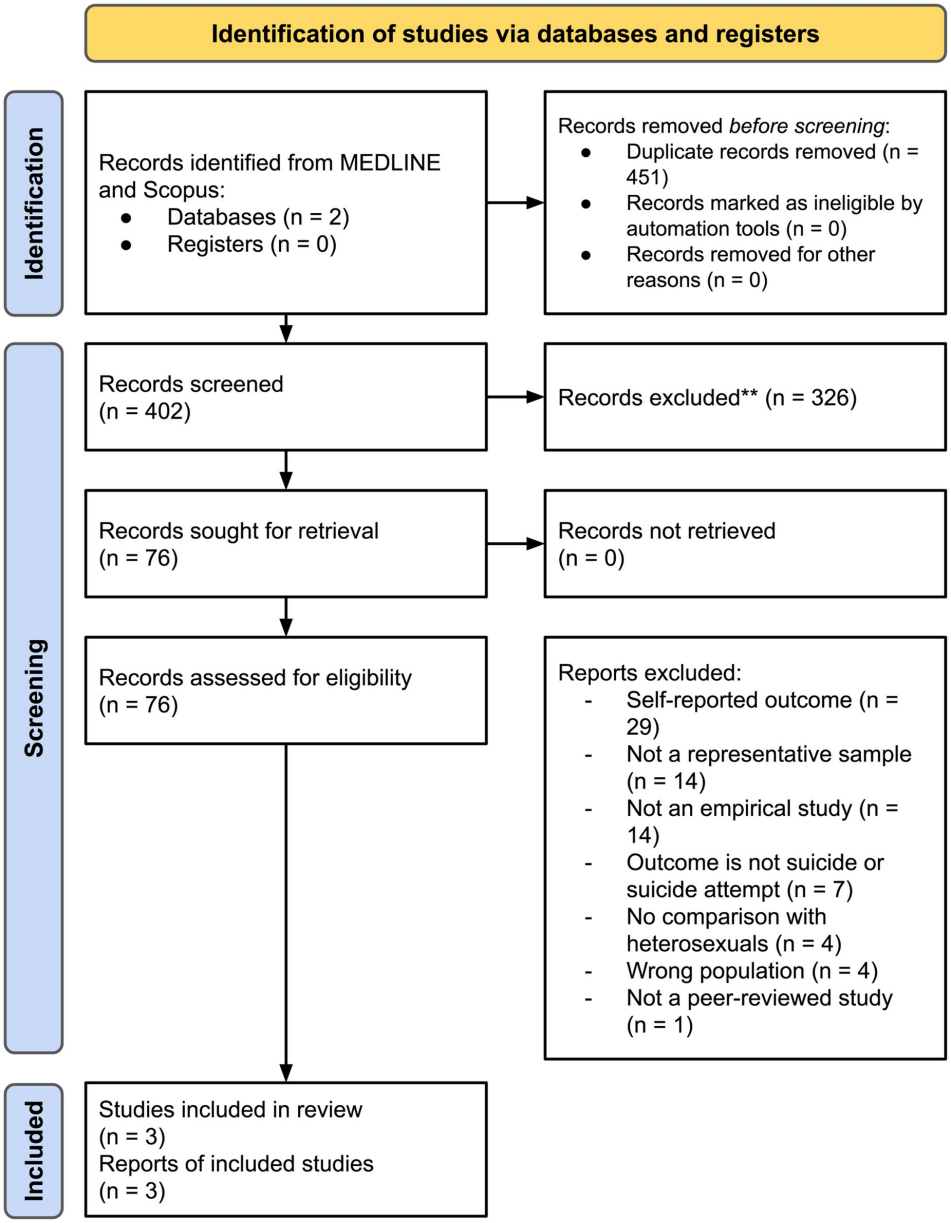
PRISMA flow diagram and search terms for literature search. Search terms of: (“lesbian” OR “gay” OR “bisexual” OR “sex*” OR “minorit*” OR “orientation” OR “homosexual” OR “sex* identity”) AND (“suicid*” OR “self?harm”) AND (“administrative” OR “medical?records” OR “hospital-based” OR “clinical” OR “emergency” OR “ED” OR “acute care”) for the period January 1995 to December 2021.

A comprehensive systematic literature search for published works from 1995 to 2021 was conducted to understand existing evidence on SRB disparity by sexual orientation and to establish the originality of this study. A broad number of search terms were used to identify studies on SRB disparities by sexual orientation that uses clinical outcomes (e.g. from health administrative records). The search terms used and PRISMA flow diagram are included in Fig 1. Out of 402 studies that were screened, only 3 studies used clinical data (i.e. not self-reported data obtained through a survey) and were based on representative samples to investigate SRB disparities by sexual orientation, however, 1) none of these studies included non-fatal SRB as a clinical outcome, 2) one American study was underpowered with only 85 sexual minority men [13], and 3) in two Danish studies [14,15]), sexual minority status was proxied by marital or partnership status, which excludes non-partnered sexual minorities and misclassifies bisexual individuals with an opposite-sex partner. Furthermore, the existing evidence on SRB disparities by sexual orientation is limited by small samples [2–5,11,16], convenience samples/selection bias [4,16–18], cross-sectional designs [1,3,5,11,16,18], and a majority of the studies rely on self-reported data [2–5,11]. These characteristics may lead to biased results [18]. There is a need for further research, using representative samples and clinical outcomes to quantify the SRB disparity by sexual orientation.

A meta-analysis of 20 cross-sectional studies (based on self-reported SRB events) found that sexual minorities report an 11–20% lifetime prevalence of suicide attempts compared to 4% in heterosexual individuals [11]. This disparity is possibly explained by the minority stress model which posits that individuals who belong to marginalized groups, including sexual minorities, experience higher stress levels and worse health outcomes due to unique social and environmental circumstances of being a member of a non-dominant group [19]. Higher levels of stress lead to psychological and biological ‘wear and tear’, subsequently leading to increased risk of SRB [20–25]. A meta-analysis found that minority stressors specific to sexual minorities, including LGBT bias-based victimization, general victimization, bullying, and negative family treatment were significantly associated with both suicidal ideation and suicide attempts among LGBT adolescents and young adults [26]. Despite general improvements in social, political, and environmental conditions, one study suggested there is still an enduring culture of homophobia, and exposure to minority stressors for sexual minorities has not decreased; the risk of SRB among sexual minorities also had not decreased, and was worse for younger sexual minority individuals [27].

Area-level material deprivation has been defined as the inability for communities to access and attain basic material needs and resources [28]. A systematic review has found that higher area-level socio-economic status (SES) is associated with lower rates of suicide in studies from North America and Europe [29]. The experiences and challenges of living in a deprived environment can result in feelings of hopelessness or despair, and increased emotional distress, which all contribute to stress and suicide among local residents, even for those who may not be deprived at an individual level [30–33]. In a study from Denmark, residents of deprived neighbourhoods had greater perceived stress than the general population, and that perceived stress was significantly associated with higher odds of health-risk behaviours related to diet, physical activity, as well as daily smoking [34]. To add, there are biological implications of the stress that comes with living in a poor area. One study assessed the allostatic load (measured by ten biomarkers of dysregulated metabolic, cardiovascular, and inflammatory systems including systolic and diastolic blood pressure) of living in deprived neighbourhoods compared to non-deprived areas: findings indicated that participants living in the most deprived quintile had 1.13 times higher allostatic load than those living in the least deprived quintile [35]. From a sociological perspective, neighbourhood deprivation may reflect the lack of neighbourhood opportunities that are health-promoting, which includes healthcare services [36], infrastructure [37], physical/built environment [38–41], and employment opportunities [42–44]. For instance, a study in Quebec found that patients from materially advantaged areas had shorter wait times to access primary care physicians compared to individuals from deprived areas, with a difference of up to 34 days for residents from the least vs. most deprived areas [36].

Minority stress theory emphasizes that experiences of discrimination and stress that sexual minorities face due to their minority status could be exacerbated by non-LGB specific stressors [45,46]. Based on evidence in the literature, there is reason to believe that environmental stressors could have a potential differential effect across sexual orientations. For example, the same level of neighbourhood environmental stressors produce different outcomes for sexual minority vs. heterosexual individuals [47–50]. In another example, a prior study showed that the effect of neighbourhood cohesion (i.e., sense of belonging and sense of shared connection with neighbours) on mental health was stronger for LGB vs non-LGB participants, suggesting that neighbourhood stressors may have a stronger influence on the health and wellbeing of LGB residents [51–54]. Overall, researchers have argued that intersectionality between SES and sexual orientation disparities deserves more attention, including contextual or area-level SES by sexual minorities [54].

There is a disproportionately high number of LGB people that live in deprived areas, who may experience additional stress due to LGB-targeted stigma [55,56]. Residents from the most deprived neighbourhoods report significantly higher levels of negative attitudes towards sexual minorities: the nationally representative Scottish Social Attitudes survey found that 25% of residents condemned same-sex relationships in deprived neighbourhoods, compared to 17% in non-deprived neighbourhoods [57]. These negative attitudes may in turn heighten sexual minorities’ risk of discrimination in deprived areas. A qualitative study found that sexual minority residents of deprived neighbourhoods experience a lack of anonymity, fear of hate crimes and aggression, and believe that their experiences would be different if they were heterosexual [12]. For example, lack of anonymity in certain deprived areas may be problematic for sexual minorities who are in the closet, if two people of the same sex were living in a single bedroom property, their sexual orientation may be assumed, and their disclosure of sexual orientation may place them in distress or danger. Due to the various ways that the effects of neighbourhood deprivation may be amplified for sexual minorities, further research is needed to examine these potential effects on SRB.

This study provides unique contributions by addressing distinct gaps in prior literature. First, as shown by our systematic search, this study contributes through the use of clinically relevant SRB outcomes. Second, we contribute by the use of a population-based sample, rather than smaller convenience samples or single-centre studies to investigate SRB by sexual orientation. Third, this is the first study to estimate the interactive effect of sexual minority status and neighbourhood deprivation on SRB. This study hypothesizes that the relationship between sexual minority status and SRB differs by level of neighbourhood material deprivation. Using a population-based survey and linked longitudinal health administrative data for people living in Ontario, this study answers the following research questions: 1) Are sexual minority status and neighbourhood deprivation independently associated with SRB risk? 2) Does neighbourhood deprivation modify the association between sexual minority status and SRB risk?

## Methods

### Data used in study cohort

Our study sample was created using Ontario participants from multiple cross-sectional cycles of the Canadian Community Health Survey (CCHS) which is linked to longitudinal health administrative datasets [58] including the Discharge Abstract Database (DAD), Ontario Mental Health Reporting System (OMHRS), National Ambulatory Care Reporting System (NACRS), and the Canada Vital Statistics Database (CVSD). The linkages between these datasets and CCHS are created and managed by Statistics Canada. DAD captures administrative, clinical and demographic information on hospital discharges (including deaths, sign-outs and transfers) [59]. NACRS contains data for hospital-based and community-based ambulatory care [60]. OMHRS reports on individuals receiving adult mental health services in Ontario, including information about mental and physical health, social supports and service use [61]. CVSD is a census of all deaths occurring in Canada each year. Deaths are reported by the provincial and territorial Vital Statistics Registries to Statistics Canada; the information provided includes demographic and cause of death information [62].

### Study participants

The study cohort is constructed by combining thirteen cycles (2003, 2005, 2007, 2008, 2009, 2010, 2011, 2012, 2013, 2014, 2015, 2016, and 2017) of the Canadian Community Health Surveys (CCHS). The CCHS is a national cross-sectional survey administered by Statistics Canada through a multistage, clustered probability sampling method. The survey collects information related to demographics, health status, healthcare use, and health determinants. Canadians aged 12 years or older can respond to the CCHS questions in either English or French by telephone through an assisted interview software. About 3% of the Canadian population could never be included in the CCHS: those living on Indigenous reserves and settlements, those living in certain remote regions of Quebec and Nunavut, full-time military members, and institutionalised individuals [63]. The CCHS has approximately 65,000 respondents per year [64]. The response rate for this survey is 75%. For our study, individuals aged 18 years and older were included. While the CCHS is cross-sectional, we built our study cohort using their linked longitudinal health administrative data for the years 2007–2017. All participants have a 2007 start time, with the following exceptions: 1) the participant was younger than 18 in 2007 (they were added the year they turn 18 years of age), and 2) among those who did not live in Ontario in 2007, they were added later once their residential address indicated an Ontario-based residence. Participants are censored if they die, or move out of province. Our study sample was constructed from 169,091 CCHS respondents weighted to represent 8,778,115 individuals and a total of 89,646,760 person-years in Ontario, Canada. As recommended by Statistics Canada, survey weights (in the form of population weights created from comparing the survey sample to the Census) were used to increase the representativeness of our sample.

### Sexual orientation

Sexual orientation was established by asking participants “Do you consider yourself to be…” and providing the following options: heterosexual, homosexual, bisexual, don’t know, and refuse to say. The single-item measure has been shown to be a valid instrument with high agreement with sexual identity (kappa statistic of 0.89) [65]. In a prior study, the CCHS question captured 99.3% of participants who identified as a sexual minority using a multi-question instrument, and 84.2% of those who reported any lifetime same-sex partners [65]. For this paper, the sexual orientation variable is divided into the following categories: heterosexual, sexual minority (gay/lesbian and bisexual), other (don’t know), and refused/not stated. A single sexual minority group was created by combining LGB respondents due to small sample size. Gender identity was not assessed in the CCHS.

### Neighbourhood deprivation

The material deprivation measure at the dissemination area (DA) level was obtained from the Ontario Marginalisation Index (ON-marg), which was created using data from Canada Census 2006, 2011, and 2016 [66,67]. ON-Marg has been used in numerous studies connecting area-level marginalization (in Ontario) with health outcomes [63,68,69]. The ON-Marg indices were joined based on historical postal code data linked by Statistics Canada. The DA has been used as a proxy for residential neighbourhoods [70–75] and it is the smallest geographic unit for which Canadian census data are available, with an average population of 400 to 700 residents [76]. The material deprivation measure was created using the following variables from the Census: proportion of the population aged 20+ without a high-school diploma, proportion of families who are single parent families, proportion of total income from government transfer payments for population aged 15+, proportion of the population aged 15+ who are unemployed, proportion of the population considered low-income, and proportion of households living in dwellings that are in need of major repair [28]. This measure of material deprivation has been found to be predictive of severe maternal morbidity in an Ontario-based study [77]. Neighbourhood deprivation is measured as an ordinal variable, ranked from 1 (least deprived) to 5 (most deprived). Each quintile group contains one fifth of the geographic units; if an area has a value of 5 on the material deprivation scale, it means it is in the most deprived 20 percent of areas in Ontario. The quintiles were created province-wide to enable comparability across the province [28].

### Outcome measure

Using linked health administrative records, the study outcome was any Ontario fatal or non-fatal SRB that resulted in hospitalisation or an emergency department visit from January 2007 to December 2017. To capture all fatal and non-fatal SRB events, SRB-related ICD-10-CA codes were extracted from the following databases: NACRS, DAD, OMHRS, and CVSD. Identification of relevant diagnostic codes was based on the definition of self-harm and suicide from the Canadian Institute of Health Information (CIHI) [78] with International Classification of Diseases version 10 (ICD-10-CA) codes: X60-X84 (intentional self-harm), Y10-Y34 (undetermined injuries), and Y87.0 (sequelae of intentional self-harm).

### Control variables

The following individual-level variables were included in the models: 1) sex (male or female), 2) age (continuous), 3) ethnic minority (no vs. yes), 4) marital status (single, unmarried, divorced/widowed), 5) chronic physical conditions, and 6) educational attainment (no highschool, highschool, bachelors, post-graduate). The Chronic Condition Indicator identifies chronic conditions including malignant cancer, diabetes, obesity, and hypertension, and was developed for use by the US Department of Health and Human Services [79]. Two area-level variables were also included in the models: 1) rurality (rural or urban by postal code), and 2) LGB density. For LGB density, the weighted proportion of sexual minorities by municipality (proxied by Census subdivision [80]) is calculated for each CCHS cycle.

### Statistical analysis

For the regression models, discrete-time survival analysis was used to assess the association between sexual minority status and SRB, and whether there is any modification by area-level material deprivation. The models compare the probabilities of experiencing an SRB event vs. not experiencing an SRB event for each person year in the study, to estimate the likelihood of having an SRB event, and examine the risk associated with the included covariates. Among the strengths of the analysis, discrete-time models are useful in studying rare events [81], including SRB [82]. We used discrete-time survival analysis since our main focus (neighbourhood deprivation) was measured on an annual basis, and to allow for area-level covariates of participants to be able to change based on residential moves and census geography changes, as the discrete analysis is better suited to include time-varying predictors using a person-year dataset which is an extension of the commonly used Cox hazards regression model [83]. Discrete-time survival analysis can be modelled through logistic regression model fit to person-year data, where the log-odds of SRB are estimated at each time point for each participant based on the predictors [84]. Our results stem from logit models using panel data with censoring, and thus the results are given in hazard odds ratios (HORs). The formula for the regression model is as follows:

$$\text{Logit}\left[\text{h}\left(\text{t}\right)/\left(1-\text{h}\left(\text{t}\right)\right)\right]\sim \text{non-time}\;\text{covariates}+\text{year}\_2007+\text{year}\_2008+\ldots +\text{year}\_2017$$

where h(t) as the hazard function is the probability of having an event during interval t.

In each person-year, participants are coded as either 1 (if they had an SRB event) or 0 (if they did not have an SRB event). If the event is fatal, then they are removed from the dataset in the following years. In the case of non-fatal SRB events, the participant will continue to be observed for further SRB events in subsequent years. Two models are presented, as we stratified by sex. Accounting for the previously mentioned covariates, the direct effects of material deprivation and sexual minority status on SRB are estimated separately for men and women. In addition, an interaction between material deprivation and sexual minority status was tested. Statistics Canada provides survey weights with the CCHS, which are applied for generalizability of the results to the Ontario population. Statistical analyses were conducted using SAS 9.4 (SAS Institute Incorporated, Cary, NC, USA), and 95% confidence intervals with two-tailed tests are used.

### Treatment of missing data

There are a few reasons loss to follow-up is not a major concern for this study. When CCHS participants agree to linkage of health records, all ED visits and hospitalizations are captured from 2007–2017 using Ontario’s complete administrative databases. Additionally, those who die or relocate to another province/country are censored. The level of missingness for the covariates are shown in Table 1 weighted sample characteristics. Individuals who did not state or did not know their sexual orientation are included as a separate category, “don’t know/not stated”, in our models. In the models, any missingness in covariates is included as a separate level to avoid loss of SRB events. Potential bias in the standard errors are accounted for through applying maximum likelihood bootstrapping with 500 replicates, which has been shown to perform similar to multiple imputations in reducing bias due to data missing at random [85].

**Table 1:** Weighted sample characteristics at baseline among men and women aged 18+ in Ontario, Canada (unweighted n = 169,090; weighted n = 8,778,120 individuals).

	Heterosexual n (%)	Sexual minority n (%)	Other n(%)	Refused/not stated n (%)	Overall n (%)
**Number of persons**	8,298,770	209,880	46,425	223,045	8,778,120
**Number of SRB events**	101,685	7,235	1,320	3,630	113,865
**Total number of person-years**	84,863,110	2,047,950	460,860	2,274,850	89,646,760
**Follow-up years**	10.226	9.758	9.927	10.199	10.213
**Mean age as of 2007 (standard deviation)**	42.12 (0.02)	38.13 (0.61)	47.79 (2.05)	51.69 (0.51)	42.3 (0.02)
**Sex**					
Male	4,074,210 (49.1%)	103,480 (49.3%)	1,7630 (38.0%)	114,145 (51.2%)	4,309,465 (49.1%)
Female	4,224,560 (50.9%)	106,400 (50.7%)	28,795 (62.0%)	108,900 (48.8%)	4,468,655 (50.9%)
**Educational attainment**					
No highschool	736,745 (8.9%)	15,130 (7.2%)	8,500 (18.3%)	57,180 (25.6%)	817,550 (9.3%)
Highschool	215,8755 (26.0%)	55,530 (26.5%)	14,810 (31.9%)	68,570 (30.7%)	2,297,660 (26.2%)
Post-secondary	4,533,415 (54.6%)	112,615 (53.7%)	17,800 (38.3%)	73,870 (33.1%)	47,37,695 (54.0%)
Post-graduate	80,1165 (9.7%)	25,235 (12.0%)	2,040 (4.4%)	12,110 (5.4%)	840,545 (9.6%)
Missing	68,695 (0.8%)	1,375 (0.7%)	3,280 (7.1%)	11,315 (5.1%)	84,665 (1.0%)
**Ethnic Minority**					
Yes	2,369,435 (28.6%)	491,00 (23.4%)	20,765 (44.7%)	79,110 (35.5%)	2,518,410 (28.7%)
No	5,893,380 (71.0%)	159,705 (76.1%)	20,710 (44.6%)	133,485 (59.8%)	6,207,280 (70.7%)
Missing	35,955 (0.4%)	1,075 (0.5%)	4,950 (1.07%)	10,450 (4.7%)	52,430 (0.6%)
**Marital status**					
Single	2,250,785 (27.1%)	116,800 (55.6%)	13,775 (29.7%)	71,275 (32.0%)	2,452,635 (27.9%)
Married	5,225,820 (63.0%)	76,620 (36.5%)	24,780 (53.4%)	124,330 (55.7%)	5,451,545 (62.1%)
Divorce/widowed	812,450 (9.8%)	16,170 (7.7%)	7,635 (16.4%)	27,165 (12.2%)	863,420 (9.8%)
Missing	9,715 (0.1%)	295 (0.1%)	235 (0.5%)	275 (0.1%)	10,520 (0.1%)
**Chronic physical conditions (at least one or more)**	751,470 (9.1%)	17,980	5,005	32,630	807,085
**Proportion of LGB in census subdivision (SD)**	0.030	0.034	0.032	0.032	0.030
**Rurality**					
Urban	7,173,255 (86.4%)	190,490 (90.8%)	42,145 (90.8%)	198,165 (88.8%)	7,604,055 (86.6%)
Rural	1,125,515 (13.6%)	19,390 (9.2%)	4,280 (9.2%)	24,875 (11.2%)	1,174,060 (13.4%)
**Material deprivation**					
1 (Lowest)	2,155,040 (26.0%)	52,050 (24.8%)	9,280 (20.0%)	50,055 (22.4%)	2,266,430 (25.8%)
2	1,808,955 (21.8%)	43,210 (20.6%)	9,675 (20.8%)	48,595 (21.8%)	1,910,435 (21.8%)
3	1,720,615 (20.7%)	36,770 (17.5%)	10,220 (22.0%)	47,260 (21.2%)	1,814,865 (20.7%)
4	1,368,160 (16.5%)	39,635 (18.9%)	7,275 (15.7%)	40,335 (18.1%)	1,455,405 (16.6%)
5 (Highest)	1,153,480 (13.9%)	36,430 (17.4%)	9,455 (20.4%)	35,475 (15.9%)	1,234,835 (14.1%)
Missing	92,525 (1.1%)	1,780 (0.8%)	515 (1.1%)	1,325 (0.6%)	96,145 (1.1%)

## Results

### Baseline characteristics

All results are reported with the CCHS survey weights applied. The baseline characteristics of the sample, stratified by sexual minority status are included in Table 1. Out of the 89,646,760 person-years, there were 113,865 SRB events: there were 7,235 events among sexual minorities and 101,685 events among heterosexuals. In total, approximately 4% of SRB events were fatal; however, due to privacy concerns with small cell sizes, we are unable to provide further breakdown of these events by sex, sexual minority status, and neighbourhood deprivation levels. Sexual minorities make up about 2.3% of the sample, and were younger than heterosexuals (mean age for sexual minority individuals 38.1 vs. mean age heterosexual individuals 42.3). Sexual minorities were less likely to be visible minorities (23.3% vs. 28.6%) and sexual minorities were more likely to be single (55.6% vs. 27.1%). With regards to residential context, there were more sexual minorities living in urban areas (90.8% vs. 86.4%), and more sexual minorities living in deprived neighbourhoods (17.4% vs. 13.9% in the most deprived neighbourhoods).

Table 2 presents the age-adjusted incidence rates of SRB per 100,000 person-years across the neighbourhood-deprivation quintiles from 2007–2017, stratified by sex. The table indicates that rates of SRB in male and female sexual minorities are consistently higher than heterosexuals, regardless of the level of neighbourhood deprivation. The relationship between neighbourhood deprivation and SRB approaches linearity for heterosexuals, where the lowest SRB rates are seen in the least deprived neighbourhoods, and the highest rates are in the most deprived neighbourhoods. On the other hand, in the case of sexual minorities, who may not follow socioeconomic gradients shown in the general population, the relationship appears to not be linear, where the highest SRB rates are seen in the middle quintiles of neighbourhood deprivation (i.e. Q2 for sexual minority men and Q3 for sexual minority women).

**Table 2:** Age-adjusted incidence rates of SRB across levels of neighbourhood deprivation (per 100,000 person-years) among Ontarians aged 18+ from 2007–2017, stratified by sex (unweighted n = 169,090; weighted n = 8,778,120 individuals).

	Male		Female	
	Heterosexual SRB per 100,000 person-years (95% CI)	Sexual minority SRB per 100,000 person-years (95% CI)	Heterosexual SRB per 100,000 person-years (95% CI)	Sexual minority SRB per 100,000 person-years (95% CI)
**Neighbourhood deprivation**				
Q1 (least deprived)	66 (65 to 68)	133 (116 to 149)	103 (101 to 105)	174 (158 to 189)
Q2	60 (58 to 61)	347 (324 to 369)	120 (118 to 123)	156 (143 to 169)
Q3	68 (66 to 69)	186 (168 to 205)	110 (107 to 112)	446 (414 to 479)
Q4	121 (119 to 123)	288 (363 to 413)	124 (121 to 126)	322 (301 to 342)
Q5 (most deprived)	165 (162 to 168)	252 (232 to 272)	177 (174 to 180)	329 (312 to 347)

**Fig 2:**
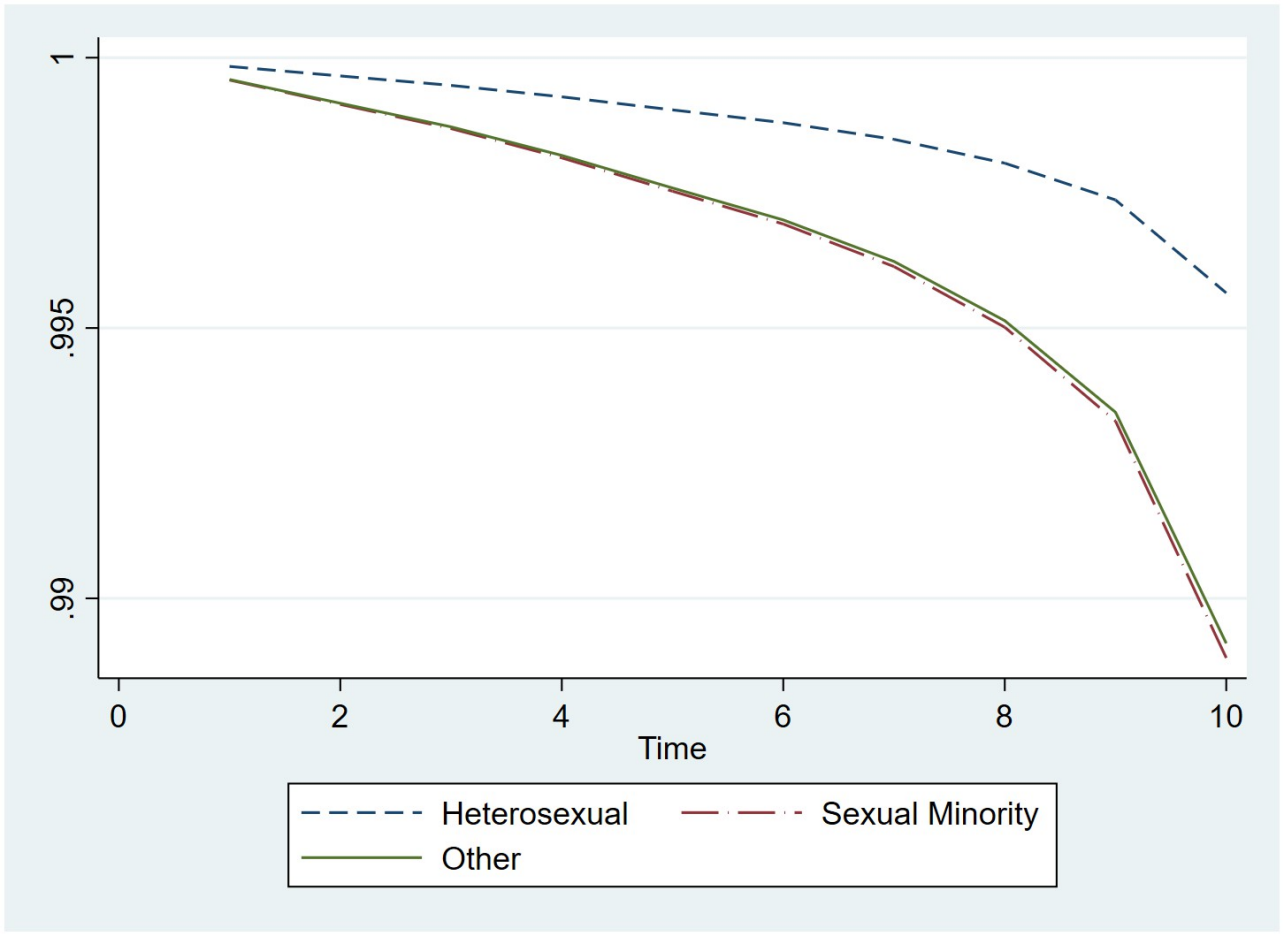
Cox regression curves modeling time to SRB event for men by sexual orientation.

**Fig 3:**
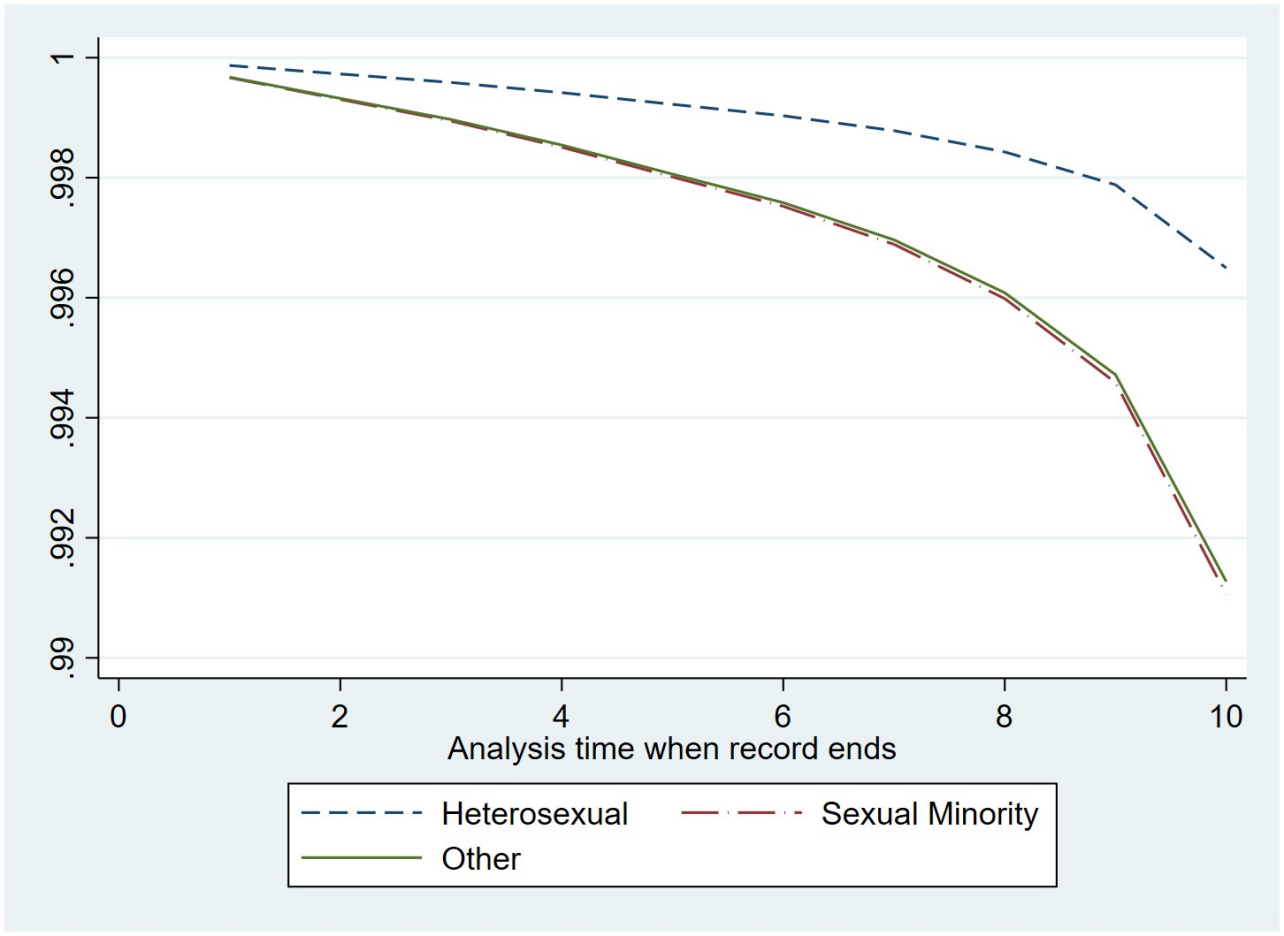
Cox regression curves modeling time to SRB event for women by sexual orientation.

Plots of survival curves (Figs 2 and 3) show that SRB events over time did not differ by sexual orientation for men and women, which provides evidence that the proportional hazard assumption was met. Table 3 presents HOR for the fully-adjusted models. The models indicate that sexual minority women had more than double the odds of SRB compared to their heterosexual counterparts (HOR: 2.149, 95% CI 1.545 to 2.989), and the odds of SRB in sexual minority men is almost three times as that of heterosexual men (HOR: 2.796, 95% CI 1.659 to 4.712). ‘Other’ (don’t know/refused to respond to the sexual orientation question) men had over three times higher odds of SRB compared heterosexual men (HOR: 3.704, 95% CI 1.716 to 7.996), while the risk for ‘other’ women was not different from the reference group.

**Table 3:** Hazard odds ratio for the risk of SRB across sexual orientation and neighbourhood deprivation among Ontarians aged 18+ from 2007–2017, stratified by sex (unweighted n = 169,090; weighted n = 8,778,120 individuals).

	Model 1: Men		Model 2: Women	
	Odds ratio (95% CI)	p-value	Odds ratio (95% CI)	p-value
**Sexual Orientation**				
Heterosexual	reference		reference	
Sexual minority	2.796 (1.659 to 4.712)	0.0001	2.149 (1.545 to 2.989)	<0.0001
Other	3.704 (1.716 to 7.996)	0.0009	0.892 (0.324 to 2.456)	0.8252
**Neighbourhood deprivation**				
Q1 (least deprived)	reference		reference	
Q2	1.097 (0.734 to 1.641)	0.6507	1.345 (0.903 to 2.002)	0.1448
Q3	1.036 (0.708 to 1.516)	0.8550	1.130 (0.829 to 1.541)	0.4389
Q4	1.874 (1.223 to 2.872)	0.0039	1.299 (0.940 to 1.795)	0.1125
Q5 (most deprived)	2.010 (1.380 to 2.929)	0.0003	1.754 (1.282 to 2.400)	0.0004
**Ethnic Minority**				
No	reference		reference	
Yes	0.887 (0.610 to 1.290)		1.014 (0.748 to 1.375)	0.9286
**Educational attainment**				
Post-graduate	reference		reference	
Post-secondary	1.232 (0.576 to 2.638)	0.5906	1.316 (0.645 to 2.687)	0.4508
Highschool	1.522 (0.737 to 3.144)	0.2567	1.706 (0.840 to 3.465)	0.1396
No highschool	3.158 (1.501 to 6.646)	0.0025	2.466 (1.202 to 5.059)	0.0138
**Marital status**				
Married	reference		reference	
Single	1.641 (1.222 to 2.203)	0.0010	1.706 (1.317 to 2.209)	<0.0001
Divorced/widowed	2.371 (1.619 to 3.473)	<0.0001	1.727 (1.328 2.246)	<0.0001
**Chronic physical conditions**				
None	reference		reference	
At least 1 chronic condition	2.098 (1.424 to 3.091)	0.0002	2.354 (1.521 to 3.643)	0.0001
**LGB Density**	0.888 (0.149 to 5.272)	0.8959	0.235 (0.069 to 0.803)	0.0209
**Rurality**				
Urban	reference		reference	
Rural	0.913 (0.731 to 1.141)	0.4247	1.049 (0.842 to 1.307)	0.6689

The model results suggest that neighbourhood deprivation was associated with higher odds of SRB, with some evidence for dose-response effects where increased SRB risk were seen in higher levels of deprivation. For men, compared to those in the least deprived neighbourhoods (Q1), those in Q4 had 1.84 times higher odds of SRB (95% CI 1.223 to 2.872), and those in the most deprived neighbourhoods (Q5) had 2.01 times higher odds (95% CI 1.380 to 2.929). Also, women in Q5 had 1.7 times higher odds of SRB (95% CI 1.282 to 2.400). Additional models were conducted that included interaction terms between sexual minority status and neighbourhood deprivation; however, there was no evidence that the effect of neighbourhood deprivation differed across sexual orientations. Separate models (for men and women) with these interactions were also conducted with neighbourhood deprivation set as continuous variable, however there was still no evidence for effect modification.

## Discussion

This study provides evidence that sexual minorities have a greater likelihood of SRB compared to heterosexuals. ‘Other’ men (who didn’t know or refused the sexual orientation question) also exhibited elevated risk of SRB (even higher than sexual minority men), while the SRB risk for those in the ‘other’ women group was similar to heterosexual women. Further research is needed to unpack and disaggregate the ‘other’ category, which may have different meanings to men and women (or may identify with them for different reasons). The effect of neighbourhood deprivation on SRB risk was observed in sexual minority and heterosexual men and women; however, the effect of neighbourhood deprivation was not modified by sexual minority status after multivariate adjustment. While the minority stress model points to the potential for effects of socio-environmental exposures to be amplified for sexual minorities [45,46], our evidence suggests neighbourhood deprivation effects on SRB risk may be similar between heterosexuals and sexual minorities. Despite research showing that residents of deprived neighbourhoods may be less tolerant of same-sex relationships (compared to non-deprived neighbourhoods) in the Scottish context [12,57], this did not translate into heightened SRB risk in LGB residents in deprived neighbourhoods in our study. This may be due to national and cultural differences, or effect modification may only be observed in non-SRB mental health outcomes (e.g. depressive symptoms, stress, etc.), the latter of which should be tested in future research.

This paper uniquely contributes to the literature on sexual minority suicidality by providing high quality evidence through a large population-representative sample, using clinical SRB-outcomes. While prior studies have relied only on self-reported SRB outcomes, our use of clinical SRB-outcomes has the benefit of reducing the risk of selection bias (i.e. avoids loss to follow-up due to death) and response bias. Moreover, this study shows, for the first time, that at each level of neighbourhood level socio-economic status, sexual minorities had consistently higher risk of SRB compared to heterosexuals. In other words, sexual minorities are more at risk of SRB regardless of their residential context. Our results also indicate there are more sexual minorities living in the most deprived neighbourhoods (17.4%) compared to heterosexuals (13.9%), which means that even while there is no effect modification between sexual minority status and neighbourhood deprivation, a higher than expected number of sexual minorities experience these dual stressors simultaneously, which would subsequently increase the overall risk of SRB in the sexual minority group.

Our findings contribute to the existing literature by showing that elevated risk associated with sexual minority status is observed in clinical SRB outcomes, at levels in line with self-reported SRB outcomes. Compared to the elevated odds of SRB among sexual minorities in our study (2.8 times in men and 2.1 times in women), a meta-analysis of population-based longitudinal studies using self-reported outcomes found that sexual minority men had 2.21 times the odds of suicide attempts compared to heterosexual men, and sexual minority women had 1.97 times the odds of suicide attempts compared to heterosexual women [85]. The similarity of our results with prior studies of self-reported SRB may be due to the low number of fatal SRB events, which accounted for only 4% of all events in our sample.

Although there have been increasing rights and social acceptance for sexual minorities over the last 2 decades [27], there is evidence that minority stress continues to negatively impact the health of sexual minorities in Canada. This may not be a surprise, considering that after the study period, the 2018 Survey on Safety in Public and Private Spaces in Canada, that sexual minority Canadians were twice as likely as heterosexual Canadians to have reported facing harassment in public (57% vs. 22%), online (37% vs. 15%) or at work (44% vs. 22%) over the previous 12 months [86]. These numbers are an indication that there is still a need for improvement in social conditions that affect the experiences of sexual minorities.

### Neighbourhood deprivation and SRB

Our results show that individuals in the most deprived neighbourhoods had greater likelihood of SRB compared to the least deprived neighbourhoods. These results were similar to another study that used the same measure of neighbourhood deprivation and was conducted in the same province (i.e. Ontario). Using the ON-Marg index of deprivation, the study found that men aged 25 to 44 living in the most deprived neighbourhoods had 1.9 times the risk of suicide relative to men in the least deprived neighbourhoods [87], which is similar to our results (OR: 2.01, or 1.98 when converted to relative risk). For women 25 to 44 in the most deprived neighbourhoods, the risk was 1.9 times (compared to OR: 1.75, or 1.74 when converted to relative risk in our study). Our results also appear to be consistent with multilevel studies that found moderate associations between neighbourhood deprivation and SRB. For example, a study in Stockholm found a significant effect of neighbourhood deprivation on suicide attempts, with 1.11 times increased odds for each unit increase of deprivation (deprivation was measured as a continuous scale): the authors concluded that neighbourhood-level deprivation seems to have an independent effect on suicidality beyond the impact of individual characteristics [7].

### Limitations and strengths

There are limitations that need to be considered. First, the CCHS does not provide options for selecting a response to the question concerning sexual orientation that do not capture sexual minorities other than lesbian, gay, or bisexual individuals (e.g. queer). In addition, due to the rare outcome and to ensure adequate sample size for each group, sexual minorities (lesbian, gay, and bisexuals individuals) were grouped together, which prevented detecting any differences between these groups (although previous literature found that the directions of risk of attempted suicide are consistent between homosexual and bisexual groups on self-reported surveys [88]. A prior Swedish study did find more nuanced differences between homosexual and bisexual women [89]. Second, we represented sexual orientation as a time-invariant exposure. While prior literature indicated that while sexual orientation may change over time, the most likely change is in the direction of “heterosexual -> bisexual/homosexual” or “bisexual -> homosexual” [90]. Hence, sexual minorities may be misclassified as heterosexual (before they are out) which may bias the results to the null. Third, the administrative data used for the SRB outcomes can be considered a limitation in that more minor SRB events that were not presented to hospitals in Ontario were not captured. Some SRB events may have been registered with different ICD-10 codes, other than codes identified in our study. Minor events especially in areas with poor healthcare services may be under-represented (e.g. rural residents who had a SRB incident may be less likely to visit the emergency department compared to urban residents, especially if they are more physically isolated or their local hospital is understaffed); however, we included a rurality indicator to partially account for this potential confounder. Fourth, the current study is limited by survivorship bias, since only respondents who survived would be able to participate in the survey. Our study mitigates survivorship bias through its inclusion of both prospective and retrospective components. For example, a 2005 participant is part of the prospective component, as their health administrative records from 2007–2017 will detect fatal SRB, and non-fatal SRB requiring hospitalization or acute care. Additionally, any individuals who moved out of the province were not included in the full study period due to differences in administrative record keeping between provinces. Lastly, this study only used the ON-Marg measure of neighbourhood deprivation even though there are other measures of neighbourhood deprivation. However, the ON-Marg index is widely used (which improves its comparability with prior studies), and considers multiple measures of socio-economic indicators of deprivation based on the Canadian census, which offers the most reliable socio-economic data of Canadians [28]. Strengths of the study include the use of longitudinal, population-representative, clinical data with a large sample. Additionally, including both fatal and non-fatal SRB events allows for an estimate of the most severe forms of SRB that use healthcare resources in Ontario. Finally, our study contributes to the literature on neighbourhood deprivation effects on SRB by examining potential effect modification via sexual orientation.

### Policy implications

Policy changes are required to provide for more culturally informed, inclusive training of healthcare practitioners to be able to assess for suicide risk and suicidal ideation in sexual minority patients. A systematic review of interventions to reduce sexual minority stressors provides various options that may help to reduce LGB SRB across a number of settings. These interventions include anti-bullying policies in the school setting, diversity training in the workplace, and marriage-equality legislation at the state level [91]. These interventions should be considered and evaluated with SRB as an outcome in the North American context. Further research on these interventions may be also helpful in finding effective strategies to mitigate the higher risk of SRB that currently exists among sexual minorities.

Evaluations of interventions to reduce the impacts of neighbourhood deprivation on SRB risk are needed, which include programs aimed at improving neighbourhood services and opportunities (employment, education, living conditions, etc.) [92]. For instance, regional economic and transit developments that help to connect residents to employment opportunities and services may be investigated regarding its impact on SRB risk [93]. More recent studies suggest that place-based interventions may not be sufficient to address the problems associated with neighbourhood deprivation, but may be more effectively addressed through broader social policies [94]. Future research can examine effects of macro-economic policies, such as universal income [95] to tackle neighbourhood inequalities in SRB risk.

## Conclusion

Both sexual minority status and neighbourhood deprivation independently contribute to an increased risk of SRB. Given the heightened risk of SRB in sexual minorities and residents of deprived areas, further funding is needed to carry out regular screening in these communities, and healthcare workers who are situated in these communities should receive training to carry out culturally sensitive suicide prevention strategies. More research is needed 1) to evaluate interventions aimed at addressing the sexual minority SRB disparity, and 2) to evaluate the efficacy of specific interventions such as neighbourhood revitalization, and other policy changes that would ameliorate the effects of neighbourhood deprivation on SRB for both sexual minorities and the general population.
